# Study of the SARS-CoV-2 genomic data generation to evaluate the introduction of genomics in epidemiological surveillance and public health decision making

**DOI:** 10.11604/pamj.2022.41.55.32344

**Published:** 2022-01-20

**Authors:** Tiatou Souho, Lallepak Lamboni, Bianza Moise Bakadia, Essodolom Taale, Koffi Kibalou Palanga, Sabiba Kou’santa Amouzou

**Affiliations:** 1 Département des Sciences de la Vie et de la Terre, Faculté des Sciences et Techniques, Université de Kara, Kara, Togo,; 2 Department of Biomedical Engineering, College of Life Science and Technology, Huazhong University of Science and Technology, Wuhan 430074, PR China,; 3 Institut Supérieur des Métiers de l´Agriculture, Université de Kara, Kara, Togo

**Keywords:** Genomic surveillance, Africa, COVID-19, SARS-CoV-2

## Abstract

**Introduction:**

the limited number of equipped laboratories and the lack of expertise left Africa lagging behind in terms of contribution in genomic data generation. The COVID-19 pandemic has drawn the attention of all public health stakeholders so that it can be used as a marker of the efforts that public health systems can produced. The main purpose of the present analytical study was to evaluate the contribution of the African continent in the genomic surveillance of SARS-CoV-2.

**Methods:**

data from the two most popular genomic databases on SARS-CoV-2 (GISAID EpiCov and NCBI Virus) were extracted and analyzed. Comparisons were made using the sequencing ratio which represents the number of genomic sequence published over one thousands confirmed cases.

**Results:**

considering continental blocks, the Africa occupied the fourth place after Oceania, Europe and North America based on sequencing ratios. However, when the considered comparison parameter is the number of sequences, the African continent was the fifth contributor after Europe, North America, Asia and South America.

**Conclusion:**

the study showed that African countries have effectively integrated the genomic data generation in the public health response strategies but the effective use of these data for a perfect surveillance is not clearly established. There is a need for capacity building in genomic data analyses for a better response to public health threats in Africa.

## Introduction

Since its first detection in November 2019 in China, the COVID-19 has continued to spread around the world, leading to the declaration of a pandemic by the World Health Organization (WHO) in March 2020 [1]. As of August 27^th^ 2021, the disease already infected more than 214,468,601 persons and caused more than 4,470,969 deaths in the world [2].

Beyond these impressive numbers of cases and deaths, the COVID-19 presents the particularity of being regarded as a serious threat by authorities in all countries including in Africa where implementation of response programmes when dealing with public health issues is usually preceded by long advocacy periods [3-5]. Actually, Africa has long been a continent with insufficient resources as to public healthcare in terms of facilities, equipment, qualified personnel, and expertise for scientific research, which in the beginning of the pandemic raised concerns in the scientific community about the resilience of the continent towards this health crisis [6,7]. Luckily, technical, societal and economic measures have been enabled in order to fight the disease [8,9]. Such efficiency is probably the result of experience acquired during the Ebola virus outbreaks and HIV/AIDS management that likely enhanced the preparedness and response capacity in the continent especially in sub-Saharan countries, though there is still a long way to go for the response at the research level which needs to go much more faster [10,11]. Among noticeable efforts in Africa, molecular diagnostic tools have been reinforced for the virus detection and human resources have been deployed. In addition, some dedicated facilities have been built to sustain the response to the pandemic.

Genomic data discovery and sharing are determinant steps in the design of appropriate programmes against public health threats related to infectious agents [12,13]. One of the added values of genomic data collection and studies is the possibility to understand genomic dynamics. Indeed, viral mutations are responsible for the spread of different variants and hamper the effectiveness of public health interventions including diagnosis, vaccination and treatment [14,15]. Building capacities in Africa to gather genomic data on the SARS-CoV-2 and perform studies on these genomes is therefore an important approach to react in response to the pandemic. Several countries in Africa are doing their best to sequence some viral strains isolated from patients, animals or environment. Overall, African countries really afforded scientific partnerships in order to substantially contribute to global efforts for genomic studies of a virus. The purpose of the present study was to evaluate the extent at which the African scientific community participate in the SARS-CoV-2 genomic studies more than one year after the first confirmation of the disease in the continent.

## Methods

**Study design**: the present study was mainly based on data collection and analyses. Nucleotides´ sequences and sequences metadata were collected from two principal platforms: the GISAID Initiative platform [16] and the NCBI Virus platform [17]. Epidemiologic data were collected from the WHO Coronavirus (COVID-19) Dashboard [2]. All these data were extracted on August 10^th^ 2021.

**Data analysis**: metadata from both platforms were mainly used to study the geographic sources of sequences as well as the hosts from where the viral materiel was obtained for sequencing. In the present analyses, the raw number of genomic sequences (partial or complete coverage) was used to gauge the position of the African continent and its countries. The regional organisation of countries in genomic databases does not match the subdivisions in the WHO Coronavirus Dashboard, epidemiologic data were considered by countries and reorganized to allow continental comparisons. The comparison was made using a “sequencing ratio” calculated by dividing the number of published sequences of the virus isolated from human beings, environment or animals by the number of confirmed cases.

## Results

The number of sequences in both GISAID and NCBI Virus platforms is continuously growing. On August 10^th^, the precise number of sequences concerning the SARS-CoV-2 was 1064504 and 2716522 in NCBI Virus and GISAID initiative platforms, respectively. The number of records for every continent and source of virus from both platforms is presented in Table 1. The table also shows that genomic surveillance in animals and the environment is achieved in all continents except in Oceania. However, the number of sequences from animals or the environment is reduced in comparison to sequences from human hosts.

**Table 1:** number of sequences retrieved from GISAID and NCBI Virus platforms

Data source	Continent	Number of all sequences	Number of Complete genomes	Source of biological material		
				Humans	Animals	Environment
**GISAID initiative**	Europe	1591995	1547648	1590080	921	994
	North America	837020	832669	836474	375	171
	Asia	185155	179399	185036	55	64
	South America	50314	46983	50280	19	15
	Africa	33071	32563	33061	7	3
	Oceania	23619	2456	23619	0	0
	Total	2721174	2662718	2718550	1377	1247
**NCBI Virus**	Europe	622577	179848	622517	40	20
	North America	417831	215893	417672	156	3
	Oceania	13939	10301	13939	0	0
	Asia	6454	3627	6424	30	0
	Africa	2161	1290	2154	7	0
	South America	927	540	912	7	8
	Total	1063889	411499	1063618	240	31

The sequencing ratio expressed as the number of sequencing for a thousand of confirmed cases was used to evaluate the implication of genomic data generation in public health response strategies. The ratios by geographic region are presented in Table 2. Oceania scores the highest sequencing ratio; when considering the GISAID, for 1000 confirmed cases in Oceania, around 185 patients are subjected to virus isolation, virus genome sequencing and sequence submission to GISAID. In the decreasing order of sequencing ratios, Oceania is followed by Europe, North America, Africa, Asia and South America. With data from the NCBI Virus database, the order of continent is the same except that North America comes before Europe.

**Table 2:** sequencing ratios for every continent considering data from the GISAID and the NCBI Virus databases

Data source	Continent	Confirmed cases	Number of sequences from human source	Sequencing ratio (‰)
**GISAID**	Oceania	127731	23,619	184.91
	Europe	61564901	1590080	25.83
	North America	37882851	836474	22.08
	Africa	7114221	33061	4.65
	Asia	55562556	185036	3.33
	South America	41042146	50280	1.23
**NCBI**	Oceania	127731	13939	109.13
	North America	37882851	417672	11.03
	Europe	61564901	622517	10.11
	Africa	7114221	2154	0.3
	Asia	55562556	6424	0.12
	South America	41042146	912	0.02

In order to evaluate the homogeneity in the contribution of different African countries in data generation, we analyzed the number of sequences and calculated sequencing ratios for every country and results are presented in Table 3, Table 3 (suite) and Table 4. The top three countries with highest sequencing ratios are Gambia, Reunion and Mauritius, considering the GISAID database. Several African countries did not publish their sequences in the NCBI database. From those present in the NCBI Virus database, the highest ratios were obtained for Djibouti, Sierra Leone and Egypt. Egypt was the only African country with sequences from animal hosts. Environment-isolated virus sequences were reported for Malawi and Morocco.

**Table 3:** sequencing ratios of African countries considering data from the GISAID database

Country	Confirmed cases	Number of submissions on GISAID	Complete genome coverage	Source of biological material			sequencing ratio (‰)
				Human	Animal	Environment	
Gambia	8763	687	682	687			78.40
Reunion	40245	2469	2469	2469			61.35
Mauritius	5219	249	249	249			47.71
Equatorial Guinea	8951	191	190	191			21.34
Burkina Faso	13626	264	264	264			19.37
Angola	43747	784	783	784			17.92
Congo	13293	227	227	227			17.08
DR Congo	51985	870	869	870			16.74
Djibouti	11663	139	139	139			11.92
Kenya	212573	2392	2392	2392			11.25
Ghana	108226	1113	1105	1113			10.28
Guinea-Bissau	4788	48	48	48			10.03
Guinea	27112	250	250	250			9.22
Central African Republic	11174	100	99	100			8.95
Benin	9065	80	80	80			8.83
Gabon	25487	219	99	219			8.59
Senegal	68012	522	516	522			7.68
Togo	16946	129	129	129			7.61
Malawi	56135	369	369	368		1	6.56
Uganda	95875	600	600	600			6.26
Rwanda	76645	474	474	474			6.18
Botswana	130771	762	762	762			5.83

**Table 3 (suite):** sequencing ratios of African countries considering data from the GISAID database

Country	Confirmed cases	Number of submissions on GISAID	Complete genome coverage	Source of biological material	sequencing ratio (‰)		
				Human	Animal	Environment	
Sierra Leone	6315	36	36	36			5.70
Nigeria	178508	994	974	994			5.57
South Africa	2540222	13721	13,584	13721			5.40
South Sudan	11139	60	60	60			5.39
Zimbabwe	116853	561	558	561			4.80
Niger	5682	24	24	24			4.22
Mozambique	133177	489	489	489			3.67
Egypt	284789	1016	994	1009	7		3.54
Zambia	200201	692	556	692			3.46
Madagascar	42781	122	122	122			2.85
Côte d’Ivoire	51399	145	145	145			2.82
Cameroon	82064	217	212	217			2.64
Mali	14648	36	36	36			2.46
Somalia	15939	33	33	33			2.07
Namibia	121203	231	230	231			1.91
Chad	4980	9	9	9			1.81
Comoros	4031	6	6	6			1.49
Lesotho	13843	18	18	18			1.30
Cabo Verde	34078	40	40	40			1.17
Eswatini	31738	33	33	33			1.04
Morocco	701325	330	319	328		2	0.47
Tunisia	613628	132	125	132			0.22
Algeria	182368	35	24	35			0.19
Ethiopia	284531	25	25	25			0.09
Libya	269847	22	22	22			0.08

**Table 4:** sequencing ratios of African countries considering data from the NCBI Virus database

Country	Confirmed cases	Number of sequences on NCBI	Complete genome coverage	Human source sequences	Sequencing ratio
Djibouti	11663	123	123	123	10.55
Sierra Leone	6315	66	57	66	10.45
Egypt	284789	825	763	818	2.87
Ghana	108226	297	185	297	2.74
Kenya	212573	503	14	503	2.37
Benin	9065	12	12	12	1.32
Zimbabwe	116853	98	0	98	0.84
Gambia	8763	6	6	6	0.68
Gabon	25487	17		17	0.67
Togo	16946	11	5	11	0.65
Guinea	27112	13	2	13	0.48
Somalia	15939	5	5	5	0.31
Uganda	95875	28	14	28	0.29
Libya	269847	46	17	46	0.17
Mali	14648	2	2	2	0.14
Tunisia	613628	77	58	77	0.13
Ethiopia	284531	7	6	7	0.02
Nigeria	178508	4	2	4	0.02
Morocco	701325	15	14	15	0.02
Cameroon	82064	1		1	0.01
Zambia	200201	1	1	1	0.005
South Africa	2540222	4	4	4	0.002

## Discussion

The ongoing pandemic is generated by the spread of a previously unknown virus. The lack of information on this pathogen has lead to the multiplicity of treatment and preventive solutions that have been thus far proposed from several laboratories around de world [18]. Up to now there is no standard treatment and available vaccines still require to be well presented to populations to increase their acceptability in some regions [19]. In this context, it is important to gather maximum data about the virus in order to provide appropriate tools for the design of effective treatment and preventive approaches. One of the most useful data that should be obtained about the virus is its genome. Given the worldwide spread of the virus, it is important that every part of the world contributes to data generation. In the present study, we investigated metadata from the most popular genomic data platforms in order to determine the level of implication of the African continent in gathering these data.

Viral genomic data search platforms are accessible worldwide. In the study, we focused on the two most popular platforms (GISAID and NCBI). GISAID is the most popular database for SARS-CoV-2 sequence submissions and provides a rapid data sharing system [20]. Thus, data from this platform are mainly used to evaluate the potential for data generation in the present study. On the other hand, NCBI Virus, the most used genomic database in Africa, was explored to extract data that could give an insight on the real capacity of African institutions to actually produce and work through the whole process of genomic data generation, annotation and publication. During analyses, metadata from both platforms were considered separately because genomic data can be submitted to several databases.

As shown in Table 1, in the GISAID database, the continent that contributes with the highest number of sequences is Europe, followed by North America and Asia. When considering data from NCBI Virus, Europe and North American continents remain the major contributors. In all cases, the highest contributors are high-income countries, whereas the African continent occupies the fifth position. Hence, based on these raw data, the number of submissions seems to reflect the availability of sequencing equipments, financial resources, and qualified human resources. In order to realize a more equitable comparison, we introduced the sequencing ratio which can be considered as an index that links genomic data generation and sharing with disease burden which is represented by the number of confirmed cases (Table 2). This indicator of regional efforts to genomic data production shows that Oceania produced much more efforts with almost 185 virus isolate sequencing for every thousand confirmed cases. In Europe and North America, viral isolation and sequencing are performed 25 and 22 times for every thousand confirmed cases.

In Africa, for every thousand confirmed cases, around 5 patients undergo virus isolation, sequencing and data submission to the GISAID platform. Several African countries did not submit any sequence to the NCBI Virus database. This shows that the GISAID database is their preferred platform for genomic data submission and perhaps for further genomic explorations as well. The sequencing ratio in Africa is five folds lower than the European one and four times lower than the ratio in North America. This may be explained by the cost of the analysis, since genomic data acquisition is still expensive even with several methods having been developed for direct sequencing from clinical samples [21]. Indeed, among all the constraints that could impede the development of genomic explorations in Africa, the reduced financial resources represents the most important one. It conditions the building of scientific facilities, equipment acquisition and capacity building. The gross domestic product per capita in Africa ranges from 1660 USD in the sub-Saharan region to 3640 USD in North Africa, whereas in Europe, it ranges from 12280 USD in Eastern Europe to 46280 USD in the Western Europe [22]. Therefore, with around a ten-fold low Gross domestic product per capita, the African continent managed to perform a sequencing ratio which is only 5 times lower than the one in Europe. This underlines the investment at countries level in responding to this public health threat. Moreover, there is solidarity in generating genomic data because several African countries don´t possess DNA analysers and therefore have to send their viral isolates to laboratories in other countries for sequencing.

For an appropriate control of the pandemic, it is important to perform animal host surveillance and genomic data from viruses in animals should be produced [23]. From whole African continent, sequences of virus isolated from animals are reported only from Egypt. These sequences were obtained from *Felis catus and Canin lupus familliaris*. Environment-isolated virus sequences were only provided by two countries: Malawi and Morocco. It seems that the epidemiologic surveillance of the pandemic, at least at the genomic level, is centred in patients. This strategy could be improved by including surveillance of animal hosts for a better understanding of the virus genomic dynamics and the place of animals in the transmission and the rise of new variants.

Overall analyses performed in the course of the present study show that the COVID-19 pandemic acted as a stimulator that accelerated the genomic revolution in Africa. The continent has faced several simultaneous infectious public health threats but the genomic investigations on these infectious agents did not reach the level at which SARS-CoV-2 genomic data were generated and published. As a comparison, in less than 2 years, there are 1290 SARS-CoV-2 complete genome sequences whereas the number of complete genome sequences is 1428 for HIV-1; 11 for HIV-2 and 584 for Ebolavirus in NCBI Virus database [17]. The rapid spread of the SARS-CoV-2 and the emergence of many variants have prompted the African continent to the genomic era.

## Conclusion

The present study was mainly focused on the potential for genomic data generation. Studies on these data for the design of new diagnostic, treatment and/or preventive approaches in Africa are rare. There is a need for national, regional or even continental facilities for genomic surveillance of infectious agents and the capacity building for the development of a pool of experts that can be involved in genomic data generation as well as studying genomic data for evidence-based public health decision making.

### What is known about this topic

- *Lack of information in the potential of African countries to produce genomic data;*
- *Lack of genomic data on infectious agents*.

### What this study adds

- *COVID-19 pandemic has accelerated investments and capacity building in viruses genomic data production in Africa;*
- *African countries invested a lot in SARS-CoV-2 genomic data generation;*
- *We found that animal surveillance is an aspect that should be reinforced*.
